# Salvage of Failed Prosthetic Breast Reconstructions by Autologous Conversion With Free Tissue Transfers

**Published:** 2013-06-20

**Authors:** N. G. Rabey, K. H. Lie, D. Kumiponjera, E. Erel, J. W. Simcock, C. M. Malata

**Affiliations:** ^a^Department of Plastic and Reconstructive Surgery, Addenbrooke's University Hospital; ^b^University of Cambridge School of Clinical Medicine; ^c^Cambridge Breast Unit, Addenbrooke's University Hospital, Cambridge, UK

## Abstract

**Objective:** Implant-based breast reconstructions are conceptually simple but prone to surgical revisions. Additional procedures often fail to address the problems associated with the reconstructive outcome, especially in patients who have received radiotherapy. However, conversion to free flaps may improve symptoms and aesthetic results. We reviewed our experience in the United Kingdom with autologous replacement of failed prosthetic reconstructions with the aims of documenting the indications for “tertiary” reconstructions and comparing our outcomes with those of other centers. **Methods:** Patients undergoing salvage surgery for suboptimal prosthetic breast reconstructions between 2000 and 2012 were retrospectively reviewed for their original reconstructive operation, previous radiotherapy, indications for revision, corrective procedures undertaken, and final outcomes. **Results:** Of 14 patients identified, 7 had delayed and 7 had immediate reconstructions. Twelve had received radiotherapy; 6 before the initial delayed prosthetic reconstructions and 6 after immediate reconstructions. Ten patients presented after undergoing previous revisions of their original reconstructions (average 1.6). Indications for autologous conversion were capsular contracture, persistent pain, and poor cosmetic outcomes (often in combination). Salvage comprised explantation, total capsulectomy, and abdominal free flap reconstruction using deep inferior epigastric artery flaps (9) and transverse rectus abdominis myocutaneous flaps (5). The average interval between initial reconstruction and salvage was 8 years (*r* = 1-14). All flap transfers were successful with satisfactory aesthetic outcomes (average 21 months follow-up). **Conclusions:** We recommend early salvage autologous conversion of implant-based reconstructions once initial prosthetic reconstructions become unsatisfactory, particularly in recipients of radiotherapy. Many of these patients may have been better served by initial autologous reconstruction; the challenge is to identify them prospectively.

Postmastectomy breast reconstruction can be broadly divided into implant-based and implant-free techniques.^1^^-^4 The majority of worldwide breast reconstructions are carried out using implants because of their simplicity. These prosthetic reconstructions may occasionally include a latissimus dorsi flap.^5^^,^^6^ Implant-free reconstructions utilize pedicled flaps or free tissue transfers. The latter are harvested from the lower abdomen, buttocks, or thighs.^7^^,^^8^

Prosthetic (implant-based) reconstructions are technically simple, quick to perform, have faster recovery times and are rarely associated with significant donor site morbidity. However, breast implants are foreign bodies and carry significant risks of capsular contracture, infection, displacement, exposure, or even rupture.^1^^,^^4^^,^^9^^-^12 These events are not uncommon and the risk of complications has been shown to be more than 40% in a review of the published literature which was greatest in the context of adjuvant radiotherapy.^13^^-^15 Complications may result in implant failure, persistent pain, and unsatisfactory cosmesis, requiring revisional surgery. In a proportion of cases, recalcitrant problems necessitate multiple revisional operations which often fail to address the patients’ concerns.^16^

In contrast, autologous (implant-free) reconstructions are longer operations with a greater risk of morbidity and more protracted recovery times. Because they utilize the patient's own tissues, they provide more natural results by behaving in a more integrated manner than silicone prostheses.^17^^,^^18^ In the long-term, they are particularly durable in the face of weight fluctuations, age-related changes, and the effects of gravity. It is generally agreed that autologous flaps yield the best cosmetic results and of the options available, free-tissue transfers display the most favorable outcomes.^1^^-^3^,^^8^^,^^17^^-^19 The salvage of failed prosthetic reconstructions by conversion to autologous tissue, termed “tertiary breast reconstruction,” is therefore an attractive proposition and series have been recently reported by authors in the United States, Canada, and Belgium.^20^^-^22

We therefore decided to review our use of free tissue transfers in the salvage of failed implant-based reconstructions in the United Kingdom to document our experience and technique and better understand the indications for conversion to autologous reconstructions. This was deemed important as most patients present for such surgery after previous unsuccessful revisional operations and it may be useful to identify, in a timely manner, the subpopulation of patients that will benefit from an early switch to implant-free solutions. Lessons learned from such an exercise may be useful to other plastic surgeons, and we wanted to compare our results with the experience of others.

## PATIENTS AND METHODS

All patients referred to the senior author (C.M.M.) over a 12-year period (2000-2012) with “end-stage” or “failed” prosthetic reconstructions were identified from outpatient clinic letters and the surgeon's logbook. The cases were then studied with respect to relevant medical and surgical histories including common surgical risk factors such as smoking and obesity. Details of their original reconstructions, radiotherapy history, indications for revisional surgeries, specific free-flap salvage procedures used, and the subsequent reconstructive outcomes were recorded. All case notes were available for review.

## RESULTS

### Original prosthetic reconstructions

Fourteen consecutive patients with a median age of 50 years (*r* = 42-69) were identified for this study (Table 1). Thirteen of 14 patients had had unilateral prosthetic breast reconstructions elsewhere. Seven of the 14 original reconstructions were undertaken at the time of mastectomy (immediate), with the remaining half being delayed procedures. Eleven had had implant-only reconstructions, with the remainder having latissimus dorsi flaps combined with implants. Of the 11 implant-only procedures, 5 involved the classical expander-to-implant technique, while 6 used definitive implants at the time of reconstruction.

### Risk factors for postoperative complications

Twelve of 14 patients had received radiotherapy; 6 before the delayed reconstructions and the remaining 6 as adjuvant treatment after immediate reconstructions. Seven patients also received chemotherapy, 2 of them in the neoadjuvant setting. Only 1 of our patients had smoked regularly but had given up more than 10 years before her first operation.

### Details of revisional surgeries prior to autologous conversion

There were a variety of reasons, summarized in Table 2, which necessitated revisional surgery. Many of these complications were coexistent and had significant overlap. The types of revisional procedures they had undergone before referral to the senior author are given in Table 4.

### Indications for salvage with free flaps

The common indications for salvage were capsular contracture, intractable pain, and poor cosmesis (Table 5). When comparing Tables 2 and 5, there is a similarity in the reasons for revision and salvage, indicating that the intervening revisions between initial reconstruction and salvage did little to address the presenting complaints. The median time interval between the initial prosthetic breast reconstruction and final salvage with free tissue transfer was 8 years, with a range of 2 to 14 years.

### Salvage procedures

**Figure 1:**
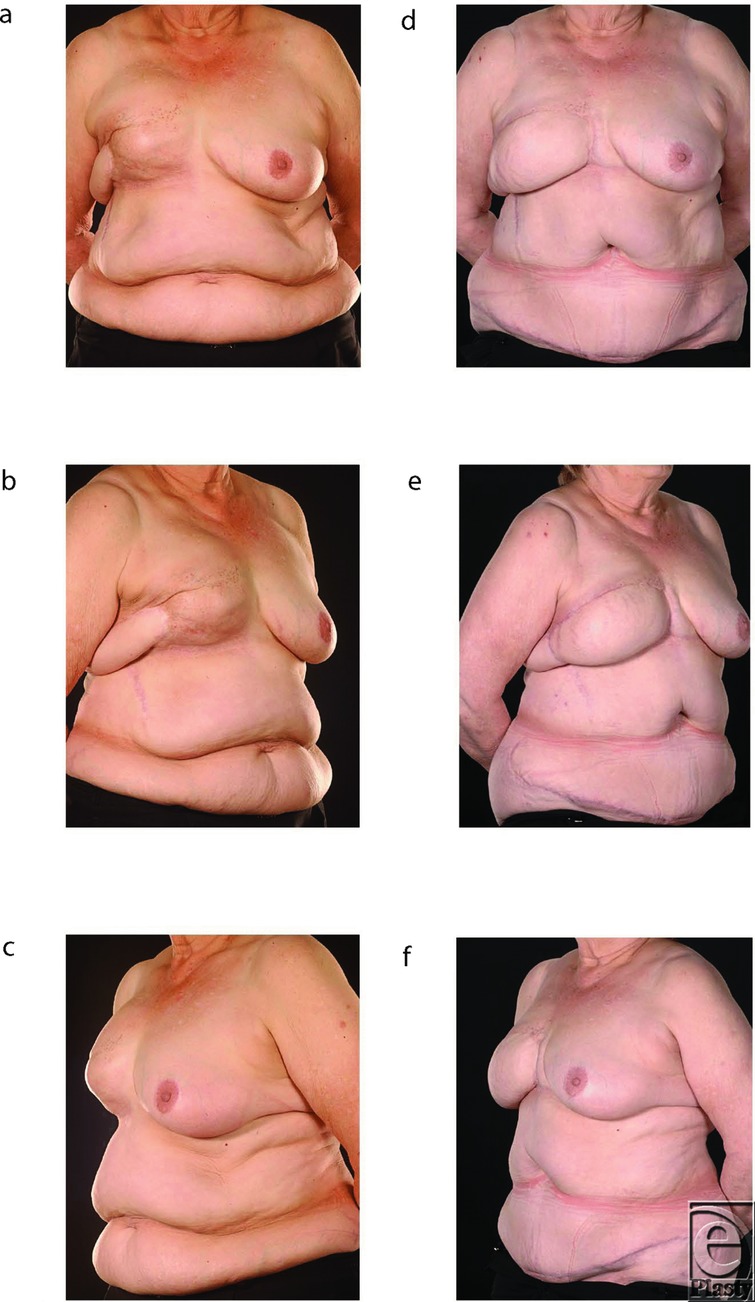
Patient 9. This 59-year-old lady with a body mass index of 38 underwent a mastectomy followed by adjuvant radiotherapy. She then had delayed expander/implant reconstruction 3 years later. This was complicated a year later by significant radionecrotic ulceration on the lateral chest wall causing implant exposure. The ulcer was treated with a local fasciocutaneous transposition flap (*a*-*c*) but she went on to develop significant capsular contracture 3 years thereafter. Despite revisional surgery, the pain and deformity persisted, and so in 2007, a free TRAM flap was performed to definitively address her persistent painful capsular contracture (*d*-*f*).

**Figure 2:**
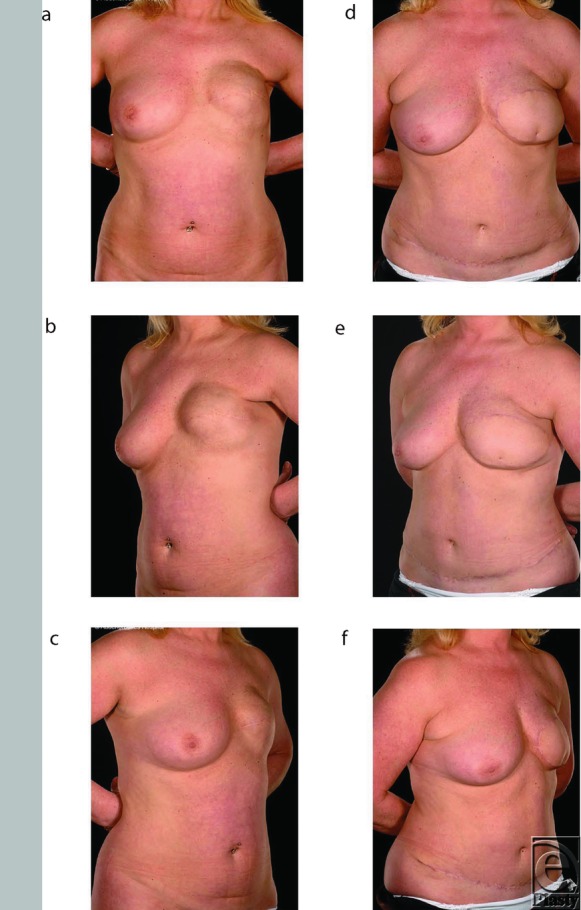
Patient 4. This 47-year-old ex-smoker had radiotherapy to her chest wall following a left mastectomy in 1997. Two years later, she was reconstructed with a Becker expander implant, which was eventually changed to a fixed-volume implant because of capsular contracture. A further exchange with a saline-filled implant still resulted in unsatisfactory cosmesis due to flatness and a poorly defined inframammary fold, along with grade IV contracture (*a*-*c*). In 2009, salvage was performed with a DIEP flap resulting in a soft and symptom-free breast (*d*-*f*).

**Figure 3:**
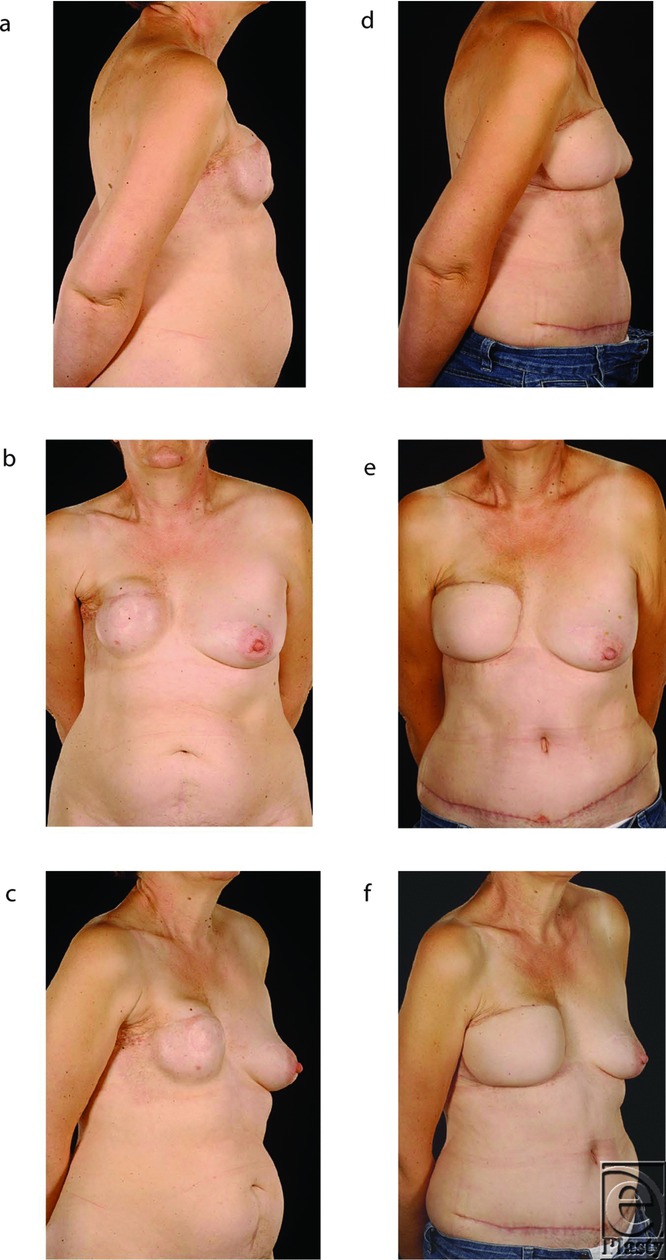
Patient 2. This 58-year-old patient had undergone a delayed right expander/implant breast reconstruction 7 years after mastectomy and adjuvant radiotherapy. She developed recurrent capsular contracture that necessitated capsulotomy and implant exchange on 2 separate occasions. However, the problem persisted over the next 5 years, and she therefore presented for a second opinion (*a*-*c*). Salvage was carried out with a contralateral free hemi-TRAM flap as she had preexisting lower midline scar. Intraoperatively, a wide resection of abnormal skin was undertaken. Her reconstructed breast is now soft, pain-free with acceptable cosmesis (*d*-*f*).

The salvage procedures consisted of total capsulectomies with explantation (Fig 1), scar releases, and creation of new pockets for the flaps in the subcutaneous (ie, the original mastectomy) plane. The free flaps used for salvage to replace the failed implant reconstructions comprised 9 deep inferior epigastric artery (DIEP) flaps (example in Fig 2) and 5 muscle-sparing free transverse rectus abdominis myocutaneous (TRAM) flaps (examples in Figs 1 and 3). They were all anastomosed end to end to the internal mammary vessels with 9/0 nylon, interrupted for the artery and continuous for the vein. Patients had an average follow-up of 21 months (*r* = 7-41) after their initial salvage procedure. All 14 patients are now asymptomatic and have acceptable cosmetic outcomes from their salvage procedures (Figs 1–3). Two free TRAM patients developed donor site hernias, which had to be repaired, and another had a Coleman fat transfer to augment her flap, which was relatively small because of her limited abdominal tissue. One patient suffered a relapse of her breast cancer 1 year after salvage that presented as phrenic nerve palsy and pulmonary metastases. All patients were happy with the cosmetic outcome and resolution of their presenting symptoms.

## DISCUSSION

Free flap salvage of failed prosthetic reconstructions constitutes a major surgical undertaking and this can be a daunting prospect for the patient. Until recently, there was little in the literature on this subject. However, a few authors have now published a series of what has come to be known as “tertiary” breast reconstruction (Table 6). Prior to these, Gurunluoglu et al^23^ described their use of deepithelialized lower abdominal free flaps for intractable capsular contracture and maintenance of breast volume in 2005 and Shestak^24^ documented satisfactory revision of similar patients with pedicled TRAM flaps. Ramakrishnan's group reported on their use of capsulotomy and bilateral DIEP flaps reconstruction in 5 patients with failed silicone implants.^25^ Grotting subsequently explained his own technique for successful use of free TRAM flaps for recalcitrant implant-related postoperative complications.^26^

Following these initial reports, other groups have reviewed their experiences in this difficult patient group.^20^^-^22 Visser et al (Canada) undertook a retrospective patient and physician assessment of the aesthetic and surgical outcomes in 42 patients undergoing tertiary breast reconstructions using free flaps.^22^ They used 47 DIEP flaps, 10 TRAM flaps, and 4 gracilis flaps in their series with 8 patients (19%) developing complications requiring reoperation. The outcomes reported by Hamdi et al^20^ (Belgium) included 81 free flaps in 54 patients, of which 81% were DIEP flaps reconstruction. They had 1 flap failure and 30 patients (55.5%) required further procedures to improve the appearance of the breast. Allen's group in the United States documented, in the largest study to date, the outcomes of 284 perforator flaps after implant failure in 191 patients.^21^ Of these, 164 were DIEP flaps reconstruction with an overall complication rate of 7.4% and 3 flap losses. However, the authors commented that the majority of patients requested implant removal with contractures of Baker I/II and no prior radiotherapy, suggesting that patients would seek autologous reconstruction for relatively minor aesthetic symptoms.

In our own experience, patients are often reluctant to undergo tertiary reconstruction, instinctively considering the process a major failure. In addition, these patients would have initially chosen implants at the time of the first reconstruction and therefore are a self-selected group less likely to favor major reconstructive surgery. Advances in radiotherapy have also reduced some of the adverse effects, many of which are at the root of these problems faced. Our unit is a university teaching hospital tertiary referral centre, and therefore this explains the large number of such cases presenting to a single surgeon.

Failed prosthetic reconstructions are a difficult problem both for the surgeon and for the patient. Most patients seeking reconstructive surgery for cosmetic sequelae are young (mostly in their forties and fifties), and they not only are concerned by the appearance of their breasts but also suffer symptoms of pain and stiffness.^27^^,^^28^ The etiology of the deformity, tightness, and associated pain stems from skin fibrosis and capsular contracture, as well as fibrosis from either repeated operations or radiotherapy. These “implant cripples” can be compared to the type 3 cosmetic sequelae described by Clough et al, in which massive retractile fibrosis of the entire reconstructed breast leads to major postoperative deformity and a suboptimal aesthetic result.^27^^,^^28^

The deformity and pain may be so severe that further revisional implant surgery is not productive, providing diminishing returns with each further operation. Hence, the equivalent of a completion mastectomy and immediate reconstruction, which would be a free-flap salvage following explantation and capsulectomy, is needed to rectify the situation. These patients differ from those undergoing the majority of the conversion operations performed by Levine et al, whereby the indications were mainly for cosmesis (request for a “natural look” in 63%). Our study is therefore more comparable to the series by Visser et al, as the common indications for the salvage surgery were physical pain, tightness, and poor cosmesis.

The decision for autologous reconstruction can be made easier in patients referred from other surgeons, as was the case in all the patients in our series. In our series, 6 of 12 patients had had at least 2 revisions following the original prosthetic reconstructions, with 3 of these 6 having 3 or more operations before final salvage. By the time the patient presents in clinic, the persistent and sometimes disabling pain may have also persuaded many of our patients to accept the more extensive surgery required to address their symptoms.

It is our opinion that a capsulectomy at the time of explantation is mandatory because it enables the creation of a virgin pocket providing a favorable bed for the flap to adhere to the underlying tissues and thus provides an improved breast shape.^25^ This is because insetting the flap into the intact, old capsule pocket would allow the flap to rotate in the smooth peri-implant space, and the smooth “mesothelial” lining would also prevent flap adherence, further compounding the problem. In fact, similar to Grotting's technique, we advocate the conversion of a subpectoral reconstruction to a subcutaneous one, as the original subpectoral reconstruction site tends to have significant amounts of scar tissue remaining, even after a total capsulectomy.^26^ The implant, capsulectomy specimen and overlying skin is always submitted for histological examination. Another reason for a total capsulectomy is that it might contribute to resolution of the pain associated with capsular contracture. Grotting postulates that the reapproximation of the pectoralis major muscle back against the chest wall contributes to the reduced pain.^26^ This is difficult to quantify. The procedure also facilitates internal mammary recipient microsurgical recipient vessel preparation.

A variable amount of skin and soft tissue resection helps to release tightness and remove damaged tissue, particularly in the case of skin, which showed visible radiation damage. In addition to the inherently poor quality of previously irradiated skin in the context of reconstructive and cosmetic outcomes, excising radiation-damaged skin also reduces the patch effect caused by the color contrast between radiation-damaged and the healthy abdominal skin of the flap. Complexion differences at the border of the skin resections can be hidden in natural lines such as the inframammary or lateral folds. This is in contrast to Ninkovic's group, who prefer to deepithelialize the free flap and preserve the damaged skin.^23^ We also find the radiotherapy-damaged skin to be thin from the previous tissue expansion and more likely to be scarred from previous surgery.

The advantages of abdominal flaps in this context are that the donor site morbidity is minimal and scarring aesthetically acceptable.^24^ Contrary to Shestak, however, our preferred abdominal flap option is a free tissue transfer as it has better vascularity than pedicled flaps and thus provides large amounts of healthy, supple skin, which is easy to reshape and provide excellent cosmesis.^19^^,^^26^ However, the contrast between healthy and irradiated skin can contribute to a marked patch effect in some patients (Fig 1). The choice of free tissue transfer is determined by the availability of tissues in the lower abdomen and by logistics as this site avoids intraoperative turning of the patient and facilitates simultaneous preparation of the recipient bed and flap harvest.^8^ The decision to perform a free muscle-sparing TRAM flap rather than a DIEP flap was undertaken in patients with borderline amounts of abdominal tissue (Figs 3a and 3b) or the presence of abdominal scars.^29^

We have demonstrated with our experience in Cambridge, United Kingdom, that salvaging failed prosthetic operations with free-tissue transfer and has provided patients with relief from their symptoms and satisfaction with the final results. Therefore, conversion of failed or suboptimal implant-based reconstructions to free flaps is certainly a viable and recommended course of action. It can also be argued that in these women it may have been preferable to have undertaken a free-tissue transfer at the time of the original procedure, rather than attempt an implant-based reconstruction, which would eventually need conversion. The challenge for the surgeon, and a potential area for future review, is therefore to recognize which patients would be more suitable for immediate autologous reconstruction so that implant failure and the need for revision remains minimal.

## CONCLUSIONS

We conclude that free tissue transfers, though a major surgical undertaking, particularly in the context of multiple previous operations, lead to satisfactory aesthetic outcomes and, more importantly for the patient, can successfully break the vicious cycle of persistent pain, poor cosmesis, and patient dissatisfaction. This surgery, however, is not to be undertaken lightly and is best undertaken in tertiary referral centers by experienced surgeons.

## Figures and Tables

**Table 1:** Summary of patients undergoing free-flap salvage of failed prosthetic reconstructions

Patient	Age (at Salvage), y	First reconstruction	Surgical risk factors	Indications*	Intervening operations	Salvage free flap	Time to flap, y	Outcome/Follow-up
1	46	Delayed LD + Becker 25	Hypertension	Poor aesthetics Pain and limited movement Implant deflation (no rupture)	…	DIEP	11	Large seroma
2	58	Delayed expander/ Implant	Pre-reconstruction radiotherapy (94Gy19#)	Poor aesthetics Baker Grade IV capsule	Capsulotomy only Implant exchange	TRAM	12	Asymptomatic
3	50	Immediate LD + Implant	Neoadjuvant chemotherapy Adjuvant radiotherapy (40Gy15#)	Poor aesthetics Radionecrosis Implant exposure	Implant Exchange	TRAM	4	Asymptomatic
4	47	Delayed Becker 25	Ex-smoker Prereconstruction radiotherapy (40Gy15#)	Poor aesthetics Baker Grade IV capsule Tightness – limited arm movement	Implant exchange ×2	DIEP	10	Asymptomatic Coleman fat transfer
5	48	Immediate implant only	Neoadjuvant chemotherapy Adjuvant radiotherapy (40Gy15#)	Poor aesthetics Pain	Capsulectomy only Implant exchange	DIEP	10	Cancer relapse 1y post salvage
6	51	Immediate expander/Implant	—	Poor aesthetics Pain	Implant exchange	DIEP	10	Asymptomatic
7	52	Delayed LD + Expander/Implant	Adjuvant chemotherapy Prereconstruction radiotherapy (40Gy15#)	Poor aesthetics Baker Grade IV capsule Chronic discharging sinus Tightness – limited arm movement	Implant exchange ×4 Division of LD flap insert Pain service	TRAM	7	Asymptomatic
8	43	Immediate implant only	Baker 4 capsule after cosmetic augmentation, converted to mastopexy Adjuvant chemotherapy Adjuvant radiotherapy (40Gy15#)	Poor aesthetics Baker Grade IV capsule (before radiotherapy) Tightness—limited arm movement	…	TRAM	2	Asymptomatic Donor site hernia repaired
9	69	Delayed implant only	Hypothyroid Prereconstruction radiotherapy (39Gy13#)	Poor aesthetics Baker Grade II capsule Significant radionecrosis	Local transposition flap Implant Exchange Pain service	TRAM	10	Asymptomatic Donor site hernia repaired
10	49	Immediate Expander/Implant	Adjuvant chemotherapy Adjuvant radiotherapy (40Gy15#)	Poor aesthetics Baker Grade IV capsule	Implant exchange	DIEP	4	Asymptomatic
11	60	Delayed implant only	Prereconstruction radiotherapy (40Gy15#)	Poor aesthetics Baker Grade IV capsule Implant rotation	Implant exchange ×3	DIEP	14	Asymptomatic
12	42	Delayed expander/Implant	Hypertension Neoadjuvant chemotherapy Prereconstruction radiotherapy (40Gy15#)	Poor aesthetics Baker Grade IV capsule Implant exposure	Implant exchange	DIEP	3	Asymptomatic
13	49	Immediate expander/Implant	Adjuvant radiotherapy Thinned out skin laterally	Poor aesthetics Baker Grade IV capsule	Implant exchange	DIEP	7	Asymptomatic
14	49	Immediate expander	superior vena cava thrombosis, neoadjuvant chemotherapy, adjuvant radiotherapy	Poor aesthetics	…	DIEP	1	Asymptomatic

*Problems encountered by patients, which necessitated the revisional operations and finally triggered the autologous salvage.LD indicates latissimus dorsi.

**Table 2:** Reconstructive complications necessitating revisional surgery

Complication	Number of patients (n = 14)
Poor cosmesis and asymmetry	14 (100%)
Recurrent/persistent pain	11 (79%)
Recurrent capsular contracture	9 (64%)
Tightness of chest wall	6 (43%)
Implant exposure/Implant rotation	3 (21%)
Radionecrotic ulcer (Fig 1a)	2 (14%)
Chronic discharging sinus	1 (7%)

**Table 3:** Additional operations to improve aesthetic outcomes

Procedure	Number of patients (n = 14)
Lipofilling	1 (7%)
Liposuction	…
Dog ear excision	…
Neobreast reduction	…
Scar revision of the breast	…
Donor site operations	2 (14%)
Nipple reconstruction (not included)	1 (7%)

**Table 4:** Types of revisional procedures performed prior to salvage

Revisional Procedure	Number of procedures*
Implant exchange	18
Capsulotomy/Capsulectomy only	2
Division of LD insertion	1
Local transposition flap	1

*Some patients had the same procedure on multiple occasions.

**Table 5:** Reconstructive complications necessitating free flap salvage

Complication	Number of patients (n = 14)
Poor cosmesis and asymmetry	14 (100%)
Recurrent/Persistent pain	12 (86%)
Recurrent capsular contracture	9 (64%)
Tightness of chest wall	6 (43%)

**Table 6:** Reported major series of tertiary breast reconstructions

Authors	Number of patients (n)	Number of (n)	DIEP (n)	Other flaps (n)	Previous radiotherapy (%)	Flap loss (%)	Revisions to improve outcome (%)	Follow-up average, mo
Hamdi et al^20^	54	81	66	15	62	1.2	55	31
Visser et al^22^	42	61	47	14	17	None	45	39
Levine et al^21^	191	284	164	120	20	1	15	NR

NR indicates not reported.
